# A New MEN2 Syndrome with Clinical Features of Both MEN2A and MEN2B Associated with a New *RET* Germline Deletion

**DOI:** 10.1155/2020/4147097

**Published:** 2020-07-29

**Authors:** Carlotta Giani, Teresa Ramone, Cristina Romei, Raffaele Ciampi, Alessia Tacito, Laura Valerio, Laura Agate, Clara Ugolini, Michele Marinò, Fulvio Basolo, Alessandro Franchi, Simona Borsari, Angela Michelucci, Cesare Selli, Gabriele Materazzi, Filomena Cetani, Rossella Elisei

**Affiliations:** ^1^Endocrine Unit, Department of Clinical and Experimental Medicine, University Hospital of Pisa, 56124 Pisa, Italy; ^2^Pathology Unit, Department of Surgical and Medical Pathology, University Hospital of Pisa, 56124 Pisa, Italy; ^3^Pathology Unit, Department of Translational Research and New Technologies in Medicine and Surgery, University Hospital of Pisa, 56124 Pisa, Italy; ^4^Unit of Molecular Genetics, Department of Laboratory Medicine, University Hospital of Pisa, 56126 Pisa, Italy; ^5^Division of Urology, Department of Translational Research and New Technologies in Medicine and Surgery, University Hospital of Pisa, 56124 Pisa, Italy; ^6^Surgery Unit, Department of Surgical and Medical Pathology, University Hospital of Pisa, 56124 Pisa, Italy

## Abstract

**Background:**

Multiple endocrine neoplasia type 2 (MEN2) is a hereditary cancer syndrome caused by *RET* proto-oncogene mutation. Two different clinical variants of MEN2 are known (MEN2A and MEN2B): medullary thyroid carcinoma (MTC) almost always present and associated with pheochromocytoma (Pheo), and primary hyperparathyroidism (HPTH) in MEN2A and with Pheo and other nonendocrine diseases in MEN2B. *Case Report*. A 7-year-old girl, previously treated for a pelvic plexiform neurofibroma, arrived at our observation with a peculiar MEN2B syndrome and with HPTH. The neck ultrasound showed bilateral thyroid nodules, local lymph node lesions, and a suspicious left hyperplastic parathyroid. The CT scan showed a megacolon and described the persistence of the pelvic tumor. A new *RET* germline deletion in exon 11 (c.1892_1899delCGAGCT; p.Glu632_Leu633del) was found. She underwent total thyroidectomy, central compartment and latero-cervical lymph node dissection, and neck exploration for primary HPTH. The histology confirmed bilateral MTC, multiple lymph node metastases, a hyperplastic parathyroid, and a parathyroid adenoma.

**Conclusions:**

This is the first case of a complex syndrome characterized by peculiar features of MEN2B, without Pheo but with a pelvic plexiform neurofibroma and with HPTH, which is typical of MEN2A. A “de novo” new germline *RET* deletion located in exon 11 was found.

## 1. Introduction

Multiple endocrine neoplasia type 2 (MEN2) is an autosomal dominant hereditary cancer syndrome caused by missense gain-of-function mutations in the *RET* proto-oncogene [1]. Medullary thyroid carcinoma (MTC) is highly penetrant in MEN2 (100% of cases), and it can be associated with pheochromocytoma (Pheo) and primary hyperparathyroidism (HPTH) [2]. Two different clinical variants of MEN2 are known: MEN2A and MEN2B. In MEN2A, approximately 50% of patients develop Pheo and less frequently (25%) develop HPTH [3]. The only nonendocrine disease that is present in 15–20% of MEN2A patients is interscapular cutaneous lichen amyloidosis (CLA) [4]. The vast majority of MEN2B patients have a marfanoid habitus, thick blueberry lips, corneal nerve thickening, and cutaneous and mucosal neuromas [5, 6]. Another very common disorder in MEN2B is gastrointestinal syndrome, varying from different degrees of constipation or, rarely, diarrhea, abdominal discomfort, and megacolon. The incidence of this disorder in MEN2B is 61–90% [7] and is due to intestinal ganglioneuromatosis, which is histologically characterized by hypertrophy of ganglion cells, supportive cells, and nerve fibers in all layers of the intestinal wall [8]. Pheo is present in approximately 50% of MEN2B cases, while HPTH has never been reported so far [6].

MEN2 syndromes are characterized by a strong genotype-phenotype correlation [1, 9]. Numerous *RET* point mutations, duplications, insertions, and deletions have been identified in patients with MEN2A, but the Cys634Arg mutation in exon 11 remains the most common mutation associated with the classic MEN2A syndrome characterized by the association of MTC with Pheo and HPTH [10]. In contrast, only two *RET* mutations (mainly M918T and, rarely, A883F) have been identified in patients with MEN2B [11]. This is the first report describing a complex syndrome in a young girl affected by MEN2B and MEN2A symptoms and carrying a *RET* deletion never described thus far in association with MEN2 syndrome (http://www.arup.utah.edu/database/MEN2/MEN2_welcome.php). Both the patient's parents and the local ethical committee gave their consent for the report of this case.

## 2. Case Presentation

A 7-year-old girl was referred to the Oncology Section of the Endocrine Unit of the University Hospital of Pisa, Italy, for suspected MTC based on the presence of both thyroid nodules and elevated serum levels of calcitonin (Ct).

The medical history of the child showed that she was born after a caesarean section at 38 weeks with a weight of 2400 g as well as with newborn jaundice and biliary vomiting. Because of the failure to pass meconium, congenital intestinal dysganglionoses or cystic fibrosis was hypothesized, but at that time, both were ruled out by a rectal biopsy and sweat test, respectively. The patient continued to experience biliary vomiting and failure to thrive; for this reason, she was fed using a nasogastric tube for a long period of time. When she was 2 years old, pyloric stenosis was suspected, and the girl underwent digestive system scintigraphy that demonstrated slowed gastric emptying. On the basis of this test, the patient underwent dilatation of the pylorus but did not achieve any clinical benefit. During gastroscopy, an irregular and small duodenum associated with esophagitis was documented. Magnetic resonance imaging (MRI) demonstrated an abdominal solid massive and expansive lesion (14 cm at the largest diameter) that extended down to the pubic region and surrounded the urethra and the bladder, without any cleavage plans, thus causing mild hydronephrosis. She underwent laparotomy with debulking of the lesion since it was unresectable. The histology described a ganglioneuroma. Over the years, she developed vesicoureteral reflux and recurrent pyelonephritis due to an increasing pressure of the pelvic mass on the bladder that forced her to practice intermittent catheterizations. At 6 years of age, urological control demonstrated high-grade (grade III) vesicoureteral reflux with markedly decreased excretive functioning bilaterally.

At 7 years of age, a serum calcium level of 11.3 mg/dl with a serum parathyroid hormone (PTH) level of 49 pg/ml was found. A neck ultrasound documented bilateral solid hypoechoic thyroid micronodules, a left roundish hypoechoic lesion of 9 mm outside the thyroid gland suspected of lymphadenopathy or hyperplastic parathyroid, and other suspected left latero-cervical lymphadenopathies. A high serum level of Ct was found (665 pg/ml Ct) with normal thyroid function. Fine-needle aspiration of the extrathyroid lesion of 9 mm was suspicious for hyperplastic parathyroid with very high levels of PTH in the wash-out of the needle used for aspiration. The fine-needle aspiration cytology (FNAC) of a left latero-cervical lymphadenopathy was diagnosed as lymph node metastasis of MTC.

**Figure 1:**
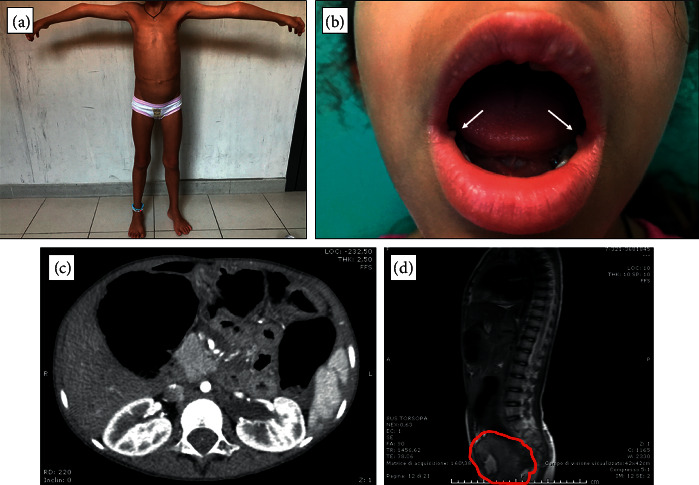
Nonendocrine features present in our patient. (a) Marfanoid habitus with long arms and long legs with respect to the trunk and skeletal abnormalities. (b) Bilateral mucosal neuromas of the mouth as indicated by the 2 arrows. (c) CT cross section of the abdomen showing the megacolon. (d) MRI sagittal section of the pelvic plexiform neurofibroma.

The patient arrived for observation in June 2018 for the management of MTC. Upon presentation, she had an evident marfanoid habitus (Figure 1(a)), and bilateral mucosal neuromas of the mouth (Figure 1(b)) that were completely overlooked until that moment.

The laboratory assessments confirmed high levels of Ct (496 ng/L; normal value <18 ng/L), normal thyroid function, hypercalcemia (11.2 mg/dL; normal range: 8.6–10.2 mg/dL), and high levels of PTH (51 ng/L; normal range: 8–40 ng/L). Moreover, an increase in bone-specific alkaline phosphatase isoenzymes (B-ALP 127 *μ*g/L; normal range: 4.7–27.1 *μ*g/L) and osteocalcin (98.7 *μ*g/L; normal range: 10.4–45.6 *μ*g/L) was found to be associated with a mild/low 25-hydroxyvitamin D level (21 *μ*g/L; normal value >30 *μ*g/L).

The neck ultrasound showed three bilateral micronodules and, in particular, a suspicious left solid hypoechoic nodule with an irregular margin of 11 mm. Below the left thyroid lobe, the hypoechoic nodular lesion of 9 mm suspicious for hyperplastic parathyroid was confirmed. In the jugular and left latero-cervical regions, multiple microlymphadenopathies were described. The total body computed tomography (CT) scan excluded the presence of distant metastases and clearly revealed the presence of a megacolon (Figure 1(c)). Examination of the pelvic mass by MRI confirmed the presence of a large pelvic lesion (Figure 1(d)) that was substantially stable with respect to the previous radiological exam.

**Figure 2:**
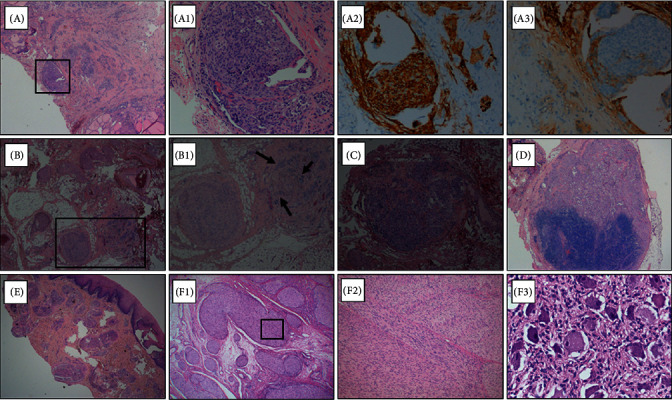
Histology of MTC and other lesions. Panels A and A_1_: MTC hematoxylin/eosin staining, magnification 2X and 10X, respectively. Panels A_2_ and A_3_: positive anticalcitonin immunostaining (10X) and negative antithyroglobulin immunostaining (10X) confirming the diagnosis of MTC. Panels B and B_1_: MTC infiltrating the perithyroid gangliar tissue, as indicated by the arrows (panel B, 2X, panel B1, 4X). Panel C: MTC vascular invasion with a neoplastic embolus (4X). Panel D: central compartment lymph node metastasis (2X). Panel E: hematoxylin/eosin staining of ganglioneuromatosis in the mucosal neuromas of the mouth excised during surgery (2X). Panel F_1_: low-power view of the pelvic plexiform neurofibroma. Panel F_2_: at higher power, the neurofibroma component consists of a uniform population of bland spindle cells with wavy elongated nuclei set in a fibrillary matrix. Panel F_3_: the ganglioneuromatosis elements, which consist of large ganglion-like neurons.

After an accurate evaluation and the exclusion of the presence of Pheo (i.e., negative adrenal gland imaging and normal values of metanephrines), she underwent total thyroidectomy, central compartment and left latero-cervical dissection, and bilateral neck exploration for primary HPTH. Moreover, during surgery, the mucosal neuromas of the mouth were biopsied for a more accurate diagnosis. The histological results described a multifocal, bilateral MTC (Figure 2, panels A and A_1_) associated with C cell hyperplasia. In particular, four different microfoci of MTC, three in the left lobe and one in the right lobe, were found; the biggest one (approximately 1 cm) was found in the left lobe, which was the only microfoci detected by neck ultrasound. A strong positive immunoreactivity to anticalcitonin (Figure 2, panel A_2_) and negative immunoreactivity to antithyroglobulin (Figure 2, panel A_3_) antibodies of the MTC tissue were shown. The largest microfocus of the tumor invaded the perithyroid soft tissue (Figure 2, panels B and B_1_), and extensive neoplastic lymphangiosis and vascular embolization (Figure 2, panel C) were present. In the perithyroid soft tissue, numerous neoplastic vascular emboli and tumoral deposits infiltrating an adjacent ganglioneuromatosis lesion were found (Figure 2, panels B and B_1_). Multiple central compartment (5/10) and left laterocervical (5/32) lymph nodes that were removed were confirmed to be metastases from MTC (Figure 2, panel D). The pathologic tumor stage according to the VIII AJCC/TNM staging [12] was pT1a(m)N1bM0. Moreover, the histology report confirmed the presence of hyperplastic parathyroid (6 mm) and parathyroid adenoma (8 mm). The histology report of the mucosal neuromas of the mouth described the presence of diffuse ganglioneuromatosis (Figure 2, panel E). Histopathologic revision of the pelvic lesion, which had been partially removed at 2 years of age in another center, revealed plexiform neurofibroma associated with ganglioneuromatosis involving the bladder wall (Figure 2, panels F_1_, F_2_, and F_3_). The plexiform neurofibroma component consisted of multiple nodules, often delimited by residual perineural sheaths (Figure 2, panel F_1_), formed by a proliferation of uniform bland spindle cells with scant cytoplasm and wavy nuclei, and set within fibrillary collagenous matrix with myxoid areas (Figure 2, panel F_2_). The ganglioneuromatosis component consisted mainly of large ganglion cells with a basophilic cytoplasm, vesicular nucleus, and prominent nucleolus (Figure 2, panel F_3_). Both components were closely related to the smooth muscle fascicles of the bladder wall. Immunohistochemical analysis revealed diffuse strong S100 positivity in the plexiform neurofibroma, whereas ganglion-like cells were positive for synaptophysin and neuron specific enolase (NSE). Epithelial membrane antigen- (EMA-) positive perineurial cells were present around the neurofibromatous fascicles.

Two months after surgery, our patient still had high levels of Ct (52 ng/L), normal calcium levels on calcitriol therapy 0.25 *μ*g/die (calcium 9.5 mg/dl; normal range: 8.6–10.2 mg/dl), normal PTH values (21 ng/L; normal range: 8–40 ng/L), and better values of both B-ALP and osteocalcin (75 *μ*g/L and 79.4 *μ*g/L, respectively). Moreover, no images suspicious for local disease recurrence were described by neck ultrasound. Eight months after surgery, the CT scan and abdominal MRI confirmed the absence of metastatic lesions and the stability of the pelvic mass. Serum Ct and CEA serum levels were also stable.

## 3. Genetic Analysis

**Figure 3:**
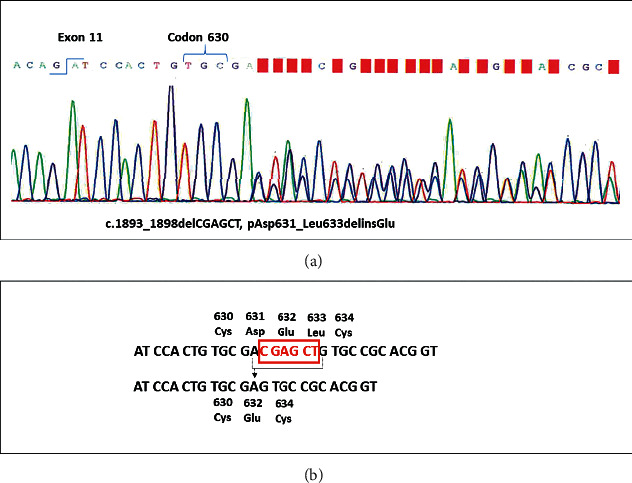
The *RET* germline deletion in exon 11 of our patient. (a) Sanger sequence analysis of the constitutive DNA showing the heterozygous germline deletion of 6 nucleotides (C GAG CT) that determined an “in frame” deletion of the *RET* gene at the level of codons 631–633 in exon 11. (b) The deletion involved the third nucleotide (i.e., C nucleotide) of codon 631, the entire codon 632, and the first two nucleotides of codon 633 (i.e., C and T nucleotides). With this deletion, codon 631 returned in frame with the third nucleotide of codon 633 (G nucleotide), changing codon 631 from aspartate (Asp) to glutamic acid (Glu).

The parents of the patient gave written informed consent for a detailed genetic examination and for the reporting of the case for scientific and/or teaching purposes. Genomic DNA was extracted from blood, amplified by PCR and sequenced as previously described [13]. A *RET* germline deletion in exon 11 involving codons 631–633 (i.e., c.1893_1898delCGAGCT; p. Asp631_Leu633delinsGlu) was found (Figure 3(a)). The deletion involved the third nucleotide (i.e., C nucleotide) of codon 631, the entire codon 632, and the first two nucleotides of codon 633 (i.e., C and T nucleotides). With this deletion, codon 631 returned in frame with the third nucleotide of codon 633 (G nucleotide), changing codon 631 from aspartate (Asp) to glutamic acid (Glu) (Figure 3(b)). An NGS approach [14] was used to analyze the MTC primary tumor, neck lymph node metastasis, and plexiform neurofibroma; no additional somatic mutations were found in the 3 tissues. Due to the complexity of the syndrome and in order to exclude the association with other genetic syndromes, such as MEN1, MEN4, neurofibromatosis 1, Cowden's disease, Marfan syndrome, and pure mucosal neuroma syndrome, we looked for mutations in the *menin*, *CDKN1B*, *NF1*, *PTEN*, *FBN1*, and *SOS1* genes, but no other variants were found.

The *RET* germline deletion was not detected in the blood of the patient's parents and brother, thus showing its “de novo” origin.

## 4. Discussion

MEN2B is a very rare syndrome in which the combination of MTC, Pheo, and some peculiar features, such as marfanoid habitus, mucosal neurinomas, and bumpy lips, are commonly associated [5]. To our knowledge, no cases of MEN2B associated with HPTH have been reported, even in large series [6]. In terms of the variance, HPTH is present in approximately 25% of MEN2A cases, but in this syndrome, the only “nonendocrine” associated disease is CLA [4]. Our patient was affected by a very peculiar syndrome characterized by the presence of the most common features of MEN2B with the simultaneous presence of HPTH, which is indeed a peculiar feature of MEN2A. Interestingly, the young patient was not affected by Pheo but by a pelvic plexiform neurofibroma with diffuse ganglioneuromatosis. Neurofibromatosis of the genitourinary tract is rare; less than 70 cases of bladder involvement, which is the most frequent site involved, have been described thus far. Among these cases, only 25 occurred in the pediatric population [15]. Plexiform neurofibroma is a benign tumor that is pathognomonic for neurofibromatosis type 1 (NF1), and it occurs in approximately 25% of people with this syndrome. To our knowledge, there are only 2 cases of reported plexiform neurofibroma with diffuse ganglioneuromatosis of the bladder in infants with NF1 [16, 17], but no cases of plexiform neurofibroma have been reported in patients with MEN2A or MEN2B.

Ganglioneuromatosis, consisting of hypertrophy of the diffuse autonomic plexus of the alimentary tract, is usually present in MEN2B and is responsible for the loss of bowel tone, distension, segmental dilation, and ultimately megacolon associated with typical gastrointestinal symptoms of MEN2B patients; however, to the best of our knowledge, no cases of ganglioneuromatosis in the urinary tract have been described in individuals with MEN2B [6]. Thus far, only two cases of adrenal ganglioneuroma in children with MEN 2 have been reported [18]. In particular, the case of a 6-year-old girl with MEN2B and an 11-year-old boy with MEN2A as well as the association with a pure single adrenal ganglioneuroma have been described. In our patient, we found a plexiform neurofibroma with diffuse ganglioneuromatosis that was a different pathological and histological entity with respect to the abovementioned cases of adrenal ganglioneuroma [19, 20].

Although germline complex alterations have already been described and reported in the ARUP database [21], the *RET* mutation found in our case has never been reported in association with MEN syndromes. Nevertheless, this variant has been previously reported at the somatic level (COSM983) [22].

Interestingly, this alteration is very close to Cys634 in exon 11. In fact, codon 634 is commonly mutated in classic MEN2A; thus, we could better expect a typical MEN2A without marfanoid habitus and mucosal neuromas that were clinically (Figure 1) and histologically (Figure 2) confirmed in our patient. Moreover, the megacolon is also typical of MEN2B but has never been reported in MEN2A. It is of interest that this *RET* mutation was a “de novo” mutation, as demonstrated by the absence of the germline mutation in the DNA of the parents and brother of our patient. In this regard, it is relevant to recall that the vast majority of MEN2B syndromes are characterized by a “de novo” mutation [6], while although possible, this event is rather rare in MEN2A [23].

Another possibility to explain this peculiar case could be the combination of MEN2B with other genetic diseases such as MEN1 or MEN4 syndromes or neurofibromatosis as well as real Marfan syndrome, but the absence of mutations in all the specific genes involved in these syndromes excluded this hypothesis.

In conclusion, this is the first case reported thus far of a complex syndrome, MEN2B/MEN2A, characterized by some peculiar features of MEN2B (i.e., MTC, marfanoid habitus and mucosal neurinomas) as well as the absence of Pheo; however, it is associated with the presence of pelvic plexiform neurofibroma, diffuse ganglioneuromatosis, and HPTH, which is indeed more characteristic of MEN2A, and is determined by a “de novo” new germline *RET* deletion located in exon 11 that is more frequently characterized by mutations correlated to MEN2A.
